# Looking out for future scientists

**DOI:** 10.7554/eLife.04901

**Published:** 2014-10-07

**Authors:** Eve Marder

**Affiliations:** Department of Biology and the Volen National Center for Complex Systems, Brandeis University, Waltham, United Statesmarder@brandeis.edu

**Keywords:** living science, careers in science, grad school, science policy, funding

## Abstract

Proposals to reduce the number of students who do PhDs are misguided, writes **Eve Marder**, because they would exclude young scientists with qualities that do not show up in exam results and interviews.

As we traveled in Greece this summer, we stumbled upon ruins from ancient times, and were reminded of the heroes, heroines, gods and goddesses who populate Greek myths and legends. In those stories, it is not uncommon for a hero to visit a seer or oracle in search of predictions of the future, often with less than salutatory consequences. Of course, seers, witches and fortune tellers figure prominently in the myths of all cultures, and queries about the future rarely result in long-term benefits to the person seeking the knowledge. That said, I wonder at those who think they can predict which of our graduate applicants is likely to become a great scientist, and am dismayed by the hubris of those who think we should restrict access to PhD programs to a select few.

**Figure 1.**
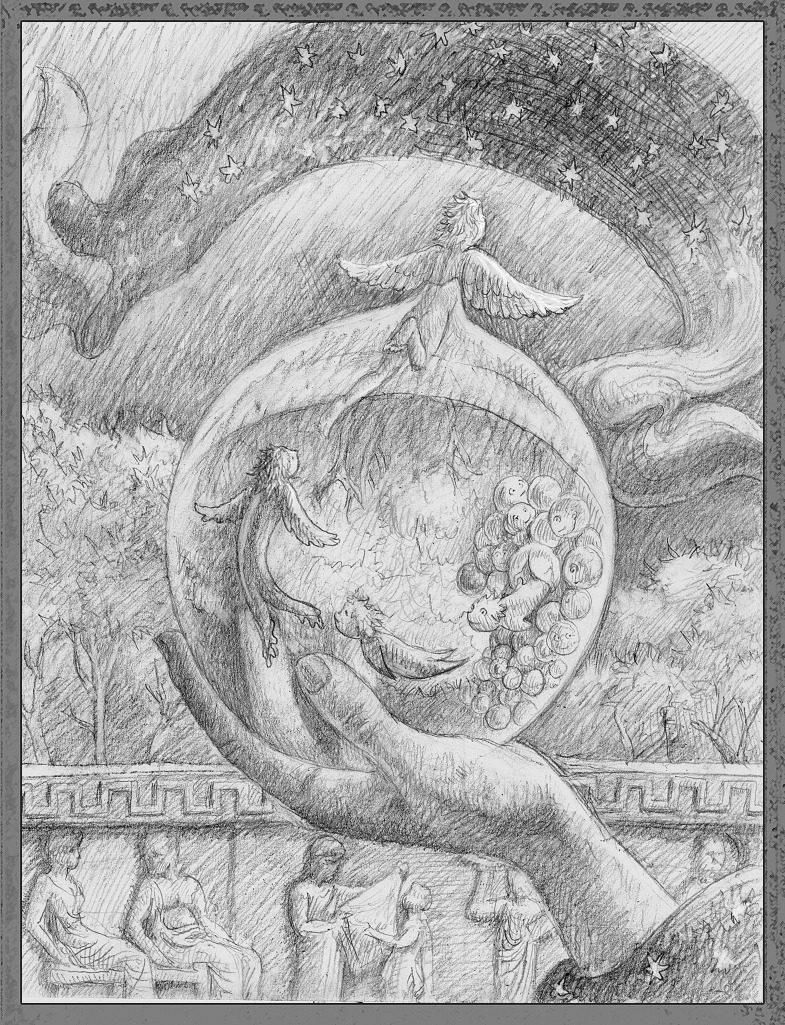
It is difficult to predict which applicants to a PhD program are likely to become outstanding scientists.

ILLUSTRATION: BEN MARDER.

Ever since I can remember (and that is a long time), there have been wise heads who have counseled that we should drastically decrease the size of our PhD classes because there are not enough academic faculty positions to accommodate all of the able and interested candidates. The recommendation was made again recently in an article by a group of four prominent researchers—Bruce Alberts, Marc Kirschner, Shirley Tilghman and Harold Varmus—that proposed a number of solutions to the various ailments that plague the biomedical work force in the US and compromise both how we do science and the joy of being a scientist (Alberts et al., 2014). Henry Bourne has made similar recommendations in *eLife* (Bourne, 2013). While these authors show a deep understanding of how increased competition for positions and funding have deleterious effects on the biomedical research and teaching enterprise, every time I think about substantially restricting access to graduate programs I wince.

I entered graduate school at a time when the Vietnam War draft opened up opportunities for women who were then thought unlikely to become successful scientists. Over the years some of our most successful students came with something that might have been considered a blemish on their records, such as years working as an artist or musician, but turned out to be an attribute that made them especially interesting, innovative or determined. Indeed, many successful scientists have told me that they would never have been admitted to graduate school with today's standards and procedures!

Many successful scientists have told me that they would never have been admitted to graduate school with today's standards and procedures!

Certainly, past performance is the best predictor of future performance, but past performance at what? Past performance in school does not necessarily select for the independent thinking and creativity that characterizes our best scientists. Past performance as a laboratory technician ensures that the candidate knows what a laboratory is, but does not necessarily mean the person will be well-suited for an academic career. But the crux of the problem is that most admissions committees will find it impossible to rank a student with straight A's lower than someone with lower scores who might have more determination, dedication and flair for science. Therefore, if we significantly decrease the number of entries into PhD programs, we risk losing some of the most exciting, dedicated and creative young scientists, and may end up with many people who have not yet made a mistake or lost their way. And, as becoming a scientist requires dealing with adversity, knowing that a prospective student has dealt with and overcome failure is probably as important as their innate intellectual gifts.

Admissions committees are bad at predicting who will end up deciding to stay in science. Even the most probing of interviews are not revealing because novice scientists often don't know how strong their curiosity, drive and ability to withstand frustration are. Some applicants with weaker paper credentials have more ability to triumph over adversity, and to keep going when the going gets tough, because they have already learned to do so. Some applicants with the strongest credentials are gifted in so many domains that they can, and do, leave PhD programs and become successful lawyers, writers, political activists and so forth. And of course, we should be proud of them as they do so!

If we knew how to spot the 25% of our applicant pool with the best combination of determination, interest and skills to become an outstanding scientist, all of the reasons articulated by Alberts et al. and Bourne might make it sensible to decrease the size of our incoming cohort. But it would be counterproductive and sad to limit our numbers and then effectively lose the creative, determined and possibly unconventional individuals who might not make it through a more restricted gateway. How do we tell the difference between the B's made by students with less talent, and those made by students who find a course boring and uninspiring, and therefore spent their time hiking, playing the violin or making films? Do students with the obedience to always do what is asked of them necessarily morph into creative and independent scientists? I suspect that many of our best scientists did not always do well in classes that they thought inane.

There are some who argue that students who finish their PhDs (or spend years as postdocs) and then move into other careers have wasted their time. I disagree. PhD students learn to think, speak and write critically. They also learn to analyze data and communicate with diverse audiences. Of course, we could devise a much shorter curriculum to enhance those skills, and probably should. Most importantly, a PhD student undertakes a difficult project to create new knowledge, and finishes it. The knowledge and confidence that come from completing an independent piece of work translates into a myriad of other life experiences. It tells the student and prospective employers that he or she has finished a difficult task, at the boundaries of what is understood about the world. Society would be enriched if more of the people making decisions in industry, law, medicine, education and politics had lived through the rigors of a PhD program, and knew first-hand how difficult it is to extract knowledge from our imperfect measurement and analytical tools.

It would be wonderful to have a crystal ball to help us know which of our applicants will blossom into a great scientist. Until then, I believe we are best served by casting our net widely, knowing that all of our graduate students will be better citizens of the world because of whatever time they spend confronting some of the deep mysteries of the universe.
